# Uptake, translocation, and phytotoxic mechanisms of polystyrene and polylactic acid nanoplastics in tobacco seedlings

**DOI:** 10.3389/fpls.2026.1878466

**Published:** 2026-07-17

**Authors:** Min-Hua Zhang, Zheng-Xiong Song, Guang-Hai Wu, Yu-Ying Xin, Zhan-Qiang Ma, Aneela Younas, Muhammad Shaaban, Yan-Fang Wang, Hong-Tao Shen, Ling Liu

**Affiliations:** 1 College of Agriculture, Henan University of Science and Technology, Luoyang, China; 2 Luoyang Branch of Henan Provincial Tobacco Corporation, Luoyang, China; 3 China Tobacco Henan Industrial Limited Company, Zhengzhou, China

**Keywords:** nanoplastics, physiological responses, polylactic acid, polystyrene, tobacco seedlings

## Abstract

Nanoplastics (NPs) are increasingly recognized as pollutants in agricultural systems, yet their impacts on tobacco remain poorly understood. This study systematically investigated the uptake, translocation, and physiological responses of tobacco seedlings exposed to polystyrene (PS, 50 nm) and polylactic acid (PLA, 50 nm) NPs under hydroponic conditions. Both PS and PLA exhibited concentration-dependent effects. Low concentrations (10 mg L^−1^) moderately enhanced biomass accumulation (8.2-24.4%) and photosynthetic performance (21.6-33.5%), whereas high concentrations (100 mg L^−1^) significantly suppressed growth (9.4-30.2%), reduced chlorophyll content (6.0-14.9%), decreased photosystem II efficiency, and increased lipid peroxidation. Confocal microscopy revealed the presence of fluorescently labeled NPs in roots, stems, and leaves, indicating their uptake and translocation within tobacco seedlings. FTIR analysis detected characteristic PS (698 cm^−1^) and PLA (1758 cm^−1^) functional groups in plant tissues and revealed changes in the -OH and C-O-C bands, indicating the occurrence of oxidative stress responses and potential modifications in celluloserelated structures. Overall, the results suggest that NPs adversely affect tobacco growth through integrated mechanisms involving tissue accumulation, redox imbalance, and structural alterations. PLA exerted stronger inhibitory and oxidative effects than PS at the same concentration. These findings advance our understanding of the behavior and phytotoxicity of biodegradable and nonbiodegradable NPs in agricultural environments.

## Introduction

Plastics, due to their low cost, high durability and versatility, are widely used in agriculture, packaging, and daily consumer products (Galahitigama et al., 2025; Yang et al., 2025). In the environment, plastics can undergo a series of biotic and abiotic processes, gradually fragmenting and aging into microplastics (MPs, particle size 1 μm-5 mm) and nanoplastics (NPs, particle size < 1 μm) (Huang et al., 2022; Rose et al., 2023). Agricultural irrigation inputs, long-term use of plastic mulching films, and the application of sludge-based fertilizers facilitate the continuous introduction and accumulation of MPs/NPs in farmland soils (Zhang et al., 2025). Compared to MPs, NPs have smaller particle sizes and exhibit stronger migration capabilities and greater potential for biological interactions in soil environments, making them more likely to come into contact with plant roots and enter plant tissues (Mitrano et al., 2021; Shen et al., 2019). Therefore, understanding the impacts of NPs on crop growth and physiological processes is crucial for evaluating agricultural ecosystems.

Currently, the effects of NPs on crops have become a research focus, and the uptake, translocation and phytotoxicity of NPs in plants have been widely studied (Azeem et al., 2022; Liu et al., 2025a). For example, exposure to polystyrene (PS) NPs has been shown to inhibit root growth, disrupt antioxidant enzyme systems, and increase lipid peroxidation in lettuce and maize (Gong et al., 2021). Similarly, amino-modified PS (PS-NH_2_) NPs significantly reduced stomatal conductance, transpiration rate, and photosynthetic capacity in rice (Zhu et al., 2023). Li et al. reported that PS NPs inhibited the growth of peanut seedlings and increased malondialdehyde content and catalase activity in leaves (Li et al., 2025). Most of these studies have focused on food crops such as corn, rice, and peanut, while research on economic crops, especially tobacco, remains limited.

Tobacco (*Nicotiana tabacum* L.) is an important economic crop in China, with its planting area and yield ranking among the top in the world (Zhu et al., 2024). Tobacco is highly sensitive to environmental stress and has a well-developed root system and a clearly defined growth cycle, making it an ideal model system for evaluating the ecological effects of NPs (Reichert et al., 2019). Although several studies have reported the effects of MPs and NPs on tobacco plants, comparative information regarding the uptake, translocation, and phytotoxic effects of biodegradable and non-biodegradable NPs in tobacco remains limited. Therefore, it is necessary to clarify the uptake and translocation characteristics of degradable and non-degradable NPs in tobacco and elucidate their underlying physiological mechanisms.

Against this background, this study selected biodegradable polylactic acid nanoplastics (PLA NPs, average particle size 50 nm) and non-biodegradable polystyrene nanoplastics (PS NPs, average particle size 50 nm), using tobacco seedlings (Zhongyan 100) as the experimental material to investigate the effects of different types and concentrations of NPs on plant growth, development, and physiological characteristics. Confocal laser scanning microscopy was employed to observe the distribution of NPs within tobacco seedlings, and Fourier transform infrared spectroscopy (FTIR) was used to analyze changes in characteristic functional groups. This study aims to elucidate the differential effects of biodegradable and non-biodegradable NPs on tobacco seedlings, thereby providing a scientific basis for understanding plant responses and potential ecological risks in agricultural ecosystems.

## Materials and methods

### Plant materials and nanoplastics

Tobacco seedlings (cv. Zhongyan 100) were obtained from Luoyang Tobacco Company, China, and raised under uniform conditions using the floating seedling method until reaching the four-leaf-one-heart stage.

Monodisperse fluorescently labeled PS nanospheres (50 nm, green fluorescence, excitation/emission: 488/520 nm) and monodisperse fluorescently labeled PLA nanospheres (50 nm, red fluorescence, excitation/emission: 632/680 nm) were obtained from Jiangsu Zhichuan Technology Co., Ltd. (China). The excitation and emission wavelengths were fixed according to the manufacturer’s specifications. Confocal laser scanning microscopy (CLSM) settings were optimized to enhance the detectable signal while avoiding photobleaching. Hoagland nutrient solution was purchased from Shanghai Macklin Biochemical Co., Ltd. (China).

### Experimental design and treatments

Tobacco hydroponic experiments were conducted at the Henan University of Science and Technology. PS and PLA NPs were ultrasonically dispersed in Hoagland nutrient solution (pH 5.5-6.5) to obtain stable suspensions at concentrations of 0, 10, and 100 mg L^-1^. Five treatments were established: CK (without NPs), PS10 (10 mg L^-1^ PS NPs), PS100 (100 mg L^-1^ PS NPs), PLA10 (10 mg L^-1^ PLA NPs), and PLA100 (100 mg L^-1^ PLA NPs). The selected concentrations were based on previous studies on the exposure level of NPs to tobacco seedlings in hydroponics (Zhang et al., 2022). Uniform and healthy tobacco seedlings were selected and pre-rinsed in ultrapure water for 24 h to remove residual substrate from the roots. Then, the tobacco seedlings were individually transferred into 100 mL conical flasks containing the corresponding treatment suspensions. Each treatment consisted of 10 independent seedlings, with one seedling cultivated per flask. The flasks were wrapped with aluminum foil to maintain root darkness. Tobacco seedlings were cultivated in a controlled growth chamber from April 25 to May 25, 2025, under a 12 h/12 h light-dark photoperiod, with day/night temperatures of 28/20 °C and relative humidity of 50%/70%. The nutrient solutions were renewed every two days to maintain consistent NP concentrations. All conical flasks were randomly arranged in the chamber and repositioned every three days to minimize positional effects. Hydroponic cultivation was employed to provide a controlled exposure environment and to facilitate the assessment of NP uptake, translocation, and phytotoxic responses without interference from soil heterogeneity. Under hydroponic conditions, NPs are readily available for plant uptake (Yildiztugay et al., 2022). Therefore, the 15-day sampling point was selected to evaluate early uptake and physiological responses, whereas the 30-day sampling point was used to assess longer-term effects.

### Determination items and methods

#### Plant growth and biomass

At 15 and 30 days after transplantation, three plants were randomly selected from the 10 biological replicates of each treatment, harvested, and separated into aboveground parts (stems and leaves) and belowground parts (roots), and the fresh weight (FW) of each fraction was measured immediately. Data are presented as mean ± standard deviation (SD) of three biological replicates.

#### Photosynthetic characteristics

Relative chlorophyll content was measured as SPAD values using a SPAD-502 chlorophyll meter (Konica Minolta, Japan). Gas exchange parameters, including net photosynthetic rate (P_n_), transpiration rate (T_r_), stomatal conductance (G_s_), and intercellular CO_2_ concentration (C_i_), were measured between 9:00 and 11:00 a.m. using a LI-6800XT portable photosynthesis system (LI-COR, USA).

#### Chlorophyll fluorescence

Chlorophyll fluorescence parameters were determined after 30 min of dark adaptation using a PAM-2100 fluorometer (Walz, Germany), including the maximum light energy conversion efficiency of PSII (F_v_/F_m_), the effective quantum yield of PSII (ΦPSII), the photochemical quenching coefficient (qP), and the non-photochemical quenching coefficient (NPQ).

#### Root morphology

Root morphological parameters (total length, average diameter, surface area, volume, tip number, and branching number) were analyzed using a WinRHIZO root scanning system (Regent Instruments, Canada).

#### Physiological and antioxidant indices

Malondialdehyde (MDA) content was determined using the thiobarbituric acid (TBA) method (Song et al., 2025). The activities of superoxide dismutase (SOD), peroxidase (POD), and catalase (CAT) were measured using the nitroblue tetrazolium (NBT) reduction method (Ismail and Hussien, 2024), the guaiacol method (Haida and Hakiman, 2019), and the ultraviolet spectrophotometry method (Sarker and Oba, 2018), respectively, and all enzyme activities were expressed on a fresh weight basis (U g^−^¹ FW).

#### Nanoplastic localization and FTIR analysis

After 30 days of exposure, fresh root, stem, and leaf tissues were harvested, rinsed with distilled water to remove surface NPs, and manually sectioned into cross-sections (thickness < 0.1 mm) using a razor blade. Sections were mounted in distilled water on glass slides with coverslips for live-tissue observation. Confocal laser scanning microscopy (CLSM; Fluo View FV3000, Olympus, Japan) was employed to visualize fluorescent NPs. Fluorescent PS NPs (50 nm, green; excitation/emission: 488/520 nm) and fluorescent PLA NPs (50 nm, red; excitation/emission: 632/680 nm) were detected under their respective excitation and emission wavelengths. To ensure consistency among samples, root tissues were prepared from the root elongation area 1 cm away from the root tip, while stem tissues were obtained from between the third and fourth functional leaves from top to bottom, and leaf tissues were collected from both sides of the middle vein of the third leaf (0.5 cm^2^ area). The same sampling positions were used for all treatments.

For FTIR analysis, dried and ground plant tissues were analyzed using a Frontier FTIR spectrometer (PerkinElmer, USA) over a spectral range of 4000–400 cm^-1^ with a resolution of 4 cm^-1^ and 16 scans per sample.

### Statistical analysis

Data were analyzed by one-way ANOVA using SPSS 21.0 (IBM, USA), followed by Duncan’s multiple range test (*P* < 0.05). All measurements were performed with three biological replicates (n = 3). Figures were prepared using Origin 2024.

## Result

### Effects of PS and PLA nanoplastics on the fresh weight of tobacco seedlings

Exposure to NPs exerted clear concentration-dependent effects on the fresh weight of tobacco seedlings (**Table 1**). At 15 days, PS10 treatment increased shoot fresh weight by 24.4%, whereas PS100 decreased shoot fresh weight by 11.9%. Similarly, PLA10 increased shoot fresh weight by 8.2%, while PLA100 markedly decreased it by 30.2%, representing the strongest inhibitory treatment. At 30 days, the overall trend was consistent with that observed at 15 days. PS10 increased shoot fresh weight by 17.7%, whereas PS100 reduced it by 9.4%. PLA10 increased shoot fresh weight by 8.9%, while PLA100 decreased it by 25.5%, indicating a sustained inhibitory effect of high-concentration PLA on shoot biomass. For root fresh weight, at 15 days, PS10 significantly increased root biomass by 25.7%, whereas PS100 showed a slight reduction of 7.6%. PLA10 increased root fresh weight by 12.3%, while PLA100 decreased by 15.7%. At 30 days, PS10 increased root fresh weight by 9.4%, whereas PS100 reduced it by 17.9%. There was no significant difference between PLA10 and CK, while PLA100 markedly decreased root fresh weight by 25.2%. Low NP concentrations promoted shoot and root growth, while high concentrations caused obvious growth inhibition, with PLA exhibiting stronger toxicity than PS.

**Table 1:** Effects of PS and PLA NPs of different concentrations on fresh weight and root morphology.

Time	Treatments	Shoot fresh weight (g)	Root fresh weight (g)	Total root length (cm)	Average root diameter (mm)	Total root surface area (cm²)	Total root volume (cm³)
15 d	CK	9.92 ± 0.41b	2.10 ± 0.18b	396.55 ± 8.50b	0.49 ± 0.01ab	53.60 ± 0.62b	1.93 ± 0.09b
	PS10	12.34 ± 1.12a	2.64 ± 0.05a	460.67 ± 6.11a	0.51 ± 0.01a	57.82 ± 0.70a	2.59 ± 0.13a
	PS100	8.73 ± 0.37c	1.94 ± 0.09c	331.37 ± 2.74 c	0.45 ± 0.10b	48.04 ± 0.82c	1.58 ± 0.11c
	PLA10	10.74 ± 0.34b	2.36 ± 0.08b	243.59 ± 5.10d	0.36 ± 0.00c	46.95 ± 1.91c	1.23 ± 0.02d
	PLA100	6.92 ± 0.29d	1.77 ± 0.07c	208.15 ± 1.95e	0.33 ± 0.01c	41.09 ± 1.61d	0.61 ± 0.05e
30 d	CK	31.64 ± 1.08c	5.52 ± 0.17b	578.33 ± 4.08ab	0.55 ± 0.06a	102.70 ± 3.81ab	5.02 ± 0.43a
	PS10	37.25 ± 0.87a	6.04 ± 0.25a	635.10 ± 11.64a	0.59 ± 0.01a	108.71 ± 8.35a	5.14 ± 0.51a
	PS100	28.65 ± 1.16d	4.53 ± 0.33c	535.44 ± 11.02b	0.46 ± 0.03b	94.27 ± 0.50bc	3.28 ± 0.22b
	PLA10	34.45 ± 0.62b	5.51 ± 0.21b	532.98 ± 48.60b	0.43 ± 0.01b	91.47 ± 4.67c	3.23 ± 0.01bc
	PLA100	23.57 ± 0.61e	4.13 ± 0.19d	459.17 ± 7.40c	0.37 ± 0.01c	83.19 ± 4.14c	2.51 ± 0.20c

The different lowercase letters indicate significant differences among different treatments at the same time (*P* < 0.05).

### Effects of PS and PLA nanoplastics on the root morphology of tobacco seedlings

NP exposure significantly affected the root morphology of tobacco seedlings (**Table 1**). At 15 days, PS10 treatment slightly promoted root elongation, with total root length increasing by 6.2% compared to CK (396.55 ± 8.50 cm), while PLA100 markedly reduced total root length by 47.5%. PS10 treatment also increased total root surface area and root volume by 7.9% and 34.2%, respectively. In contrast, all measured root morphological parameters under PLA treatments were lower than those of CK, and declined progressively with increasing concentration. At 30 days, all measured root morphological parameters increased relative to those at 15 days, but the overall response patterns remained consistent across treatments. PLA NPs strongly inhibited root elongation and expansion, exerting stronger inhibitory effects than PS. PS treatments showed a biphasic response, with stimulation at low concentrations and inhibition at high concentrations, while PLA treatments consistently exhibited inhibitory effects.

### Effects of PS and PLA nanoplastics on SPAD values of tobacco seedlings

**Figure 1:**
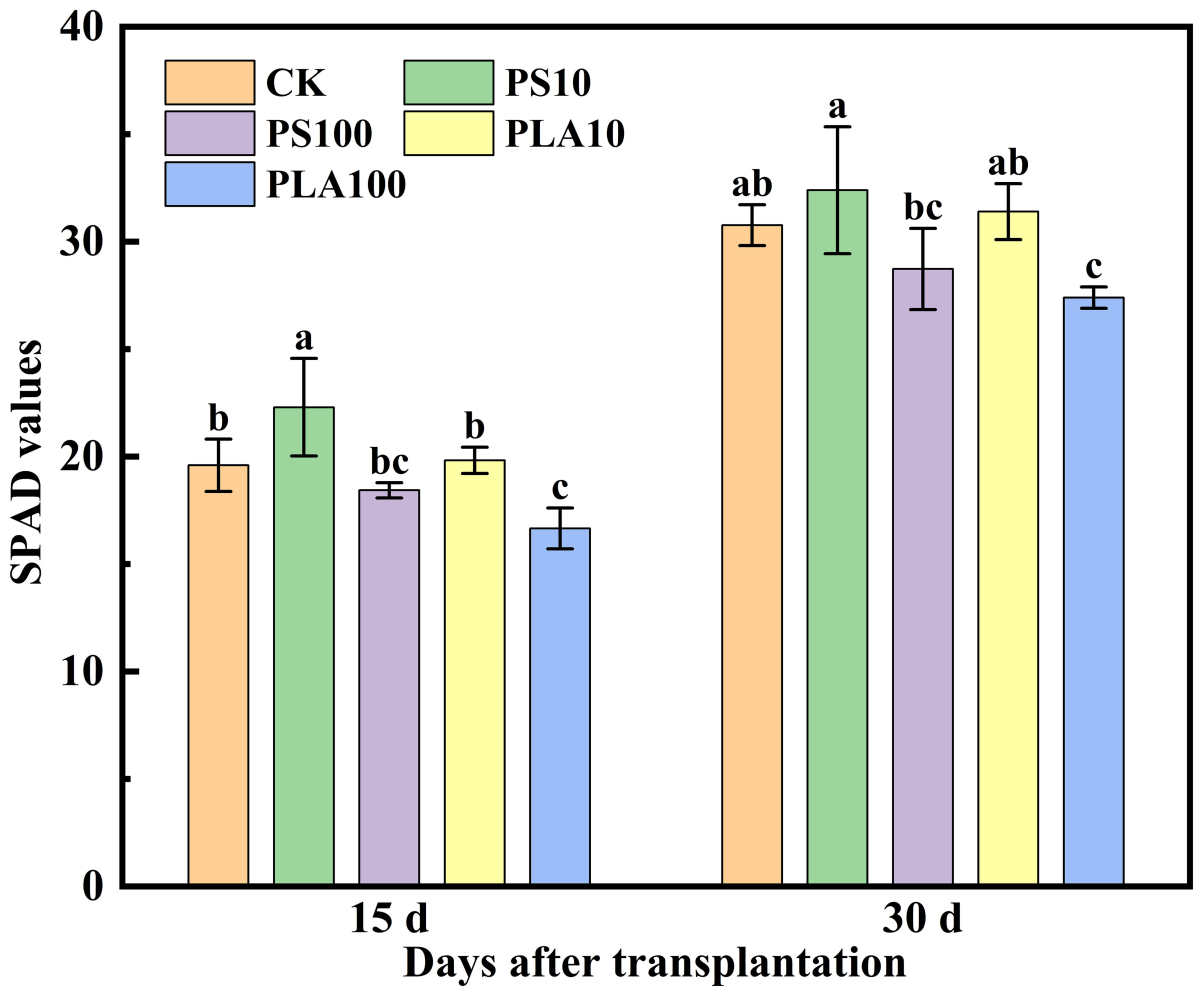
Effects of PS and PLA NPs of different concentrations on SPAD values. The bars with different lowercase letters indicate significant differences among different treatments at the same time (n=3, *P* < 0.05).

Different types and concentrations of NPs exerted distinct effects on the SPAD values of tobacco seedlings (**Figure 1**). At 15 days, compared to CK, the PS10 treatment significantly increased the SPAD value by 13.8%, indicating that low concentrations of PS NPs enhanced relative chlorophyll content. PS100 decreased the SPAD value by 6.0%, indicating an inhibitory effect at high concentrations. In contrast, PLA exhibited a weaker stimulatory effect at low concentrations, as PLA10 increased the SPAD value by only 1.2% with no significant difference from CK. However, PLA100 significantly decreased the SPAD value by 14.9%, representing the strongest inhibitory response among all treatments. These findings demonstrate a clear concentration-dependent response of chlorophyll content to NP exposure. At 30 days, SPAD values in all treatments were significantly higher than those at 15 days, reflecting the continuous accumulation of chlorophyll during plant growth and development. The low-concentration treatments (PS10 and PLA10) increased SPAD values by 5.3% and 2.1%, respectively, indicating a slight stimulatory effect. In contrast, high-concentration treatments (PS100 and PLA100) reduced SPAD values by 6.6% and 10.9%, respectively. Moreover, at the same concentration, PLA generally exerted stronger inhibitory effects than PS, showing material-specific differences in NP-induced effects on chlorophyll content in tobacco seedlings.

### Effects of PS and PLA nanoplastics on the photosynthetic characteristics of tobacco seedlings

**Figure 2:**
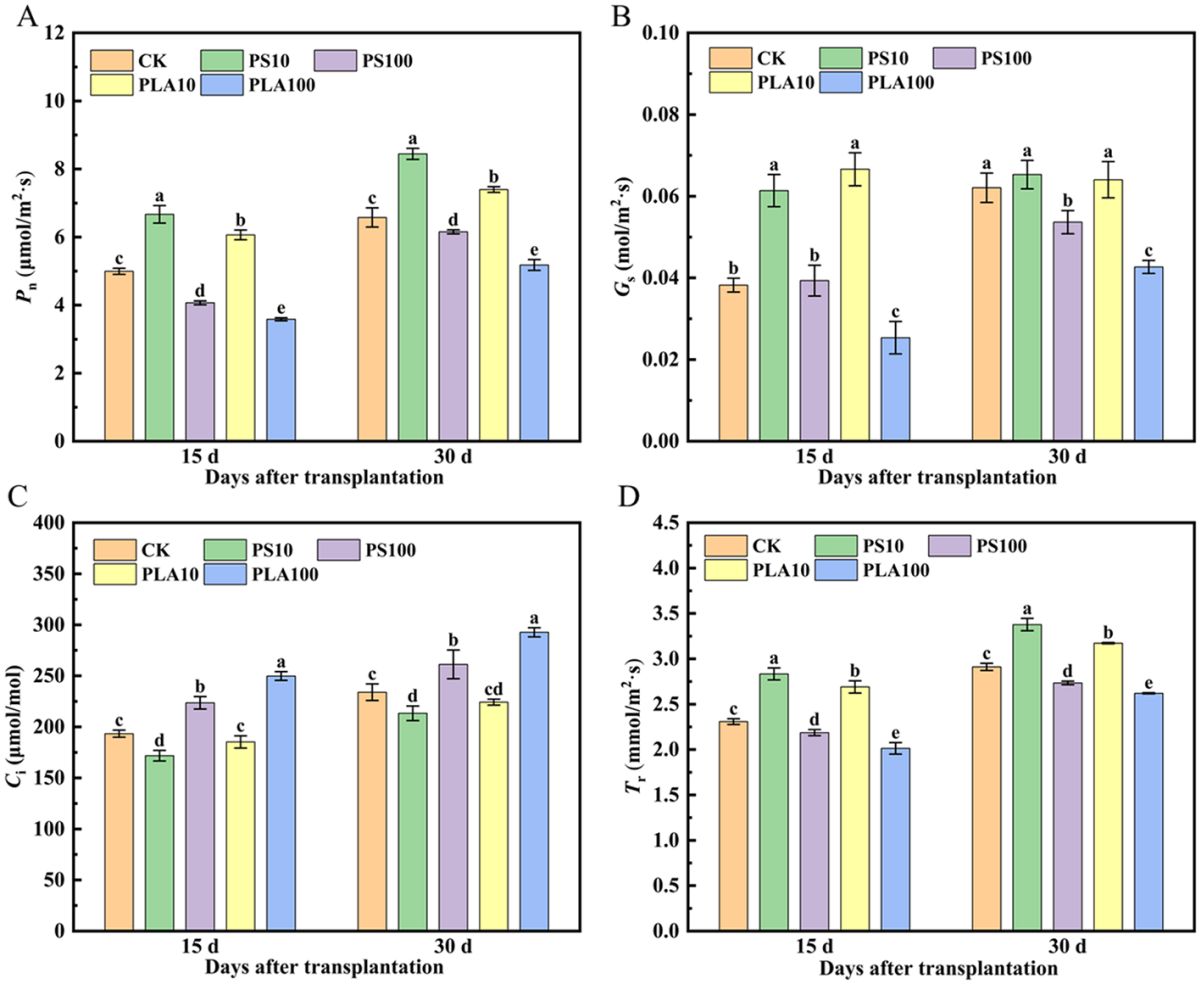
Effects of PS and PLA NPs of different concentrations on photosynthetic characteristics: **(A)** P_n_, **(B)** G_s_, **(C)** C_i_ and **(D)** T_r_. The bars with different lowercase letters indicate significant differences among the treatments (n=3, *P* < 0.05).

NP exposure significantly affected the photosynthetic characteristics of tobacco seedlings (**Figure 2**). At 15 days, compared to the control (CK), low-concentration treatments (PS10 and PLA10) promoted the net photosynthetic rate (P_n_), with PS10 and PLA10 significantly increasing P_n_ by 33.5% and 21.6%, respectively (**Figure 2A**). High-concentration treatments (PS100 and PLA100) inhibited photosynthesis, among which PLA100 caused a significant decrease of 28.3% in P_n_. At 30 days, P_n_ values generally increased across all treatments compared to those at 15 days, whereas the inhibitory effects of high-concentration treatments became more pronounced. Stomatal conductance (G_s_) and transpiration rate (T_r_) showed response patterns similar to those of P_n_ (**Figures 2B, D**). At 15 and 30 days, low-concentration treatments increased G_s_ and T_r_, while high-concentration NP treatments reduced both parameters to varying extents. The decreases were more pronounced under PLA100 treatment, indicating that NP exposure impaired stomatal regulation in tobacco leaves. The intercellular CO_2_ concentration (C_i_) exhibited an inverse pattern compared with P_n_, G_s_, and T_r_ under NP treatments (**Figure 2C**). At 15 days, C_i_ was significantly higher in the high-concentration treatments compared to CK, while no significant changes were observed under low-concentration treatments. At 30 days, a similar pattern was observed, with C_i_ remaining at relatively high levels under high-concentration treatments, particularly under PLA exposure. These results indicate that NP exposure inhibited the CO_2_ assimilation capacity in tobacco leaves.

### Effects of PS and PLA nanoplastics on the chlorophyll fluorescence of tobacco seedlings

**Figure 3:**
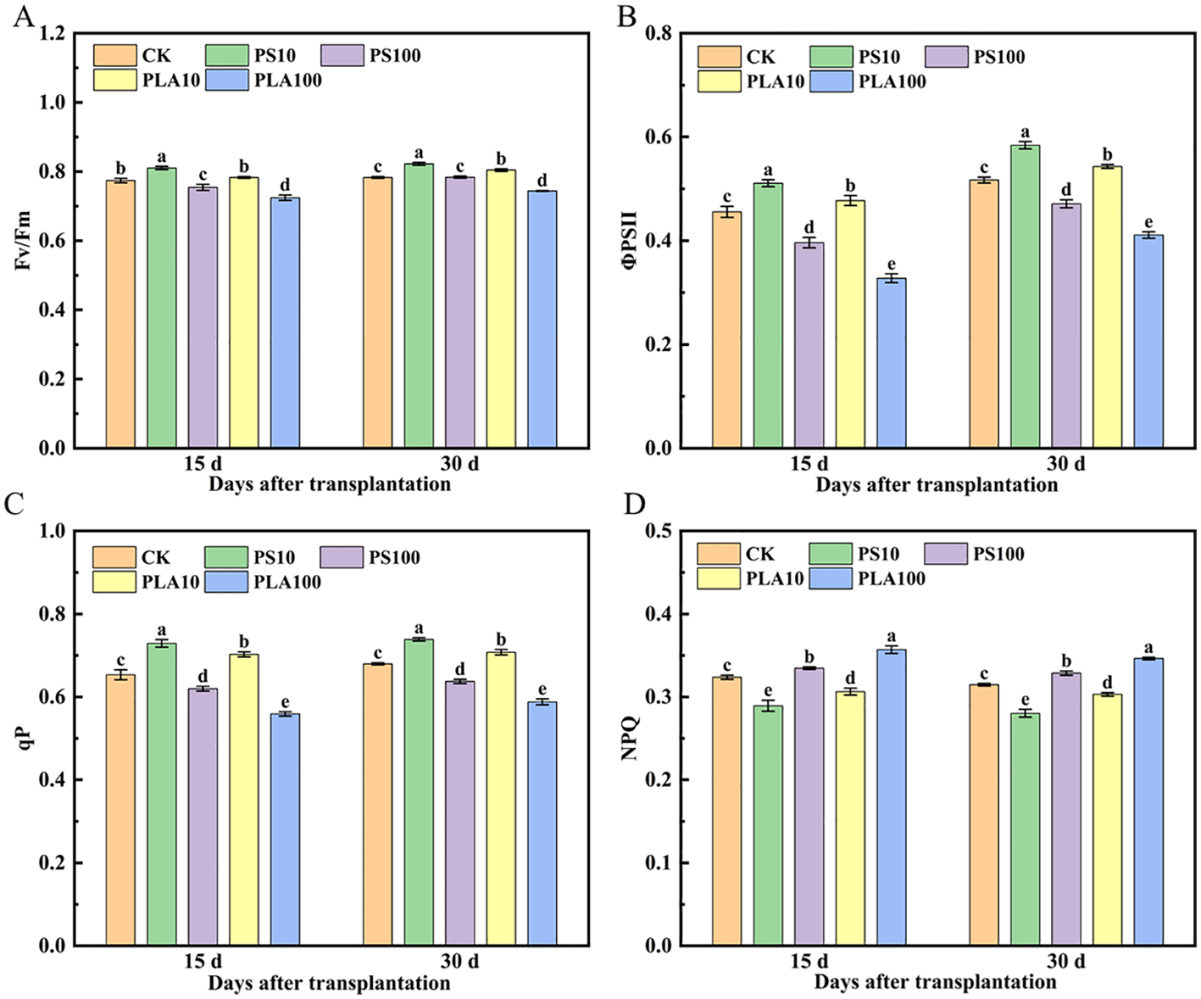
Effects of PS and PLA NPs of different concentrations on chlorophyll fluorescence: **(A)** F_v_/F_m_, **(B)** ΦPSII, **(C)** qP and **(D)** NPQ. The bars with different lowercase letters indicate significant differences among the treatments (n=3, *P* < 0.05).

NP treatments significantly affected the chlorophyll fluorescence of tobacco seedlings (**Figure 3**). At 15 and 30 days, the maximum photochemical efficiency of PSII (F_v_/F_m_) under PS10 and PLA10 did not differ significantly from CK (**Figure 3A**). PS100 and PLA100 resulted in varying degrees of F_v_/F_m_ decline, with the order of decline being: PLA100 > PS100 > CK. Compared to F_v_/F_m_, the effective quantum yield of PSII (ΦPSII) was more sensitive to NP exposure (**Figure 3B**). At 15 and 30 days, PS10 and PLA10 slightly increased ΦPSII relative to CK, whereas PS100 and PLA100 significantly decreased ΦPSII, indicating that NP stress affected the effective photosynthetic electron transport efficiency of PSII. At 15 days, the photochemical quenching coefficient (qP) increased by 11.6% (PS10) and 7.5% (PLA10) under low-concentration treatments compared to CK, while high-concentration treatments markedly decreased qP by 5.2% (PS100) and 14.4% (PLA100) (**Figure 3C**). At 30 days, no significant differences in qP were observed under low-concentration treatments, while high-concentration treatments caused further reductions of 6.2% (PS100) and 13.4% (PLA100). This reduction in qP indicates a decreased proportion of open PSII reaction centers, with the strongest inhibition observed under PLA100. The non-photochemical quenching coefficient (NPQ) exhibited an inverse trend relative to qP (**Figure 3D**). At 15 days, NPQ was significantly increased under high-concentration treatments, whereas low-concentration treatments resulted in lower NPQ compared to CK. At 30 days, NPQ remained at low levels under low-concentration treatments, while high-concentration exposure triggered a more pronounced NPQ response.

### Effects of PS and PLA nanoplastics on the antioxidant enzyme activities of tobacco seedlings

**Figure 4:**
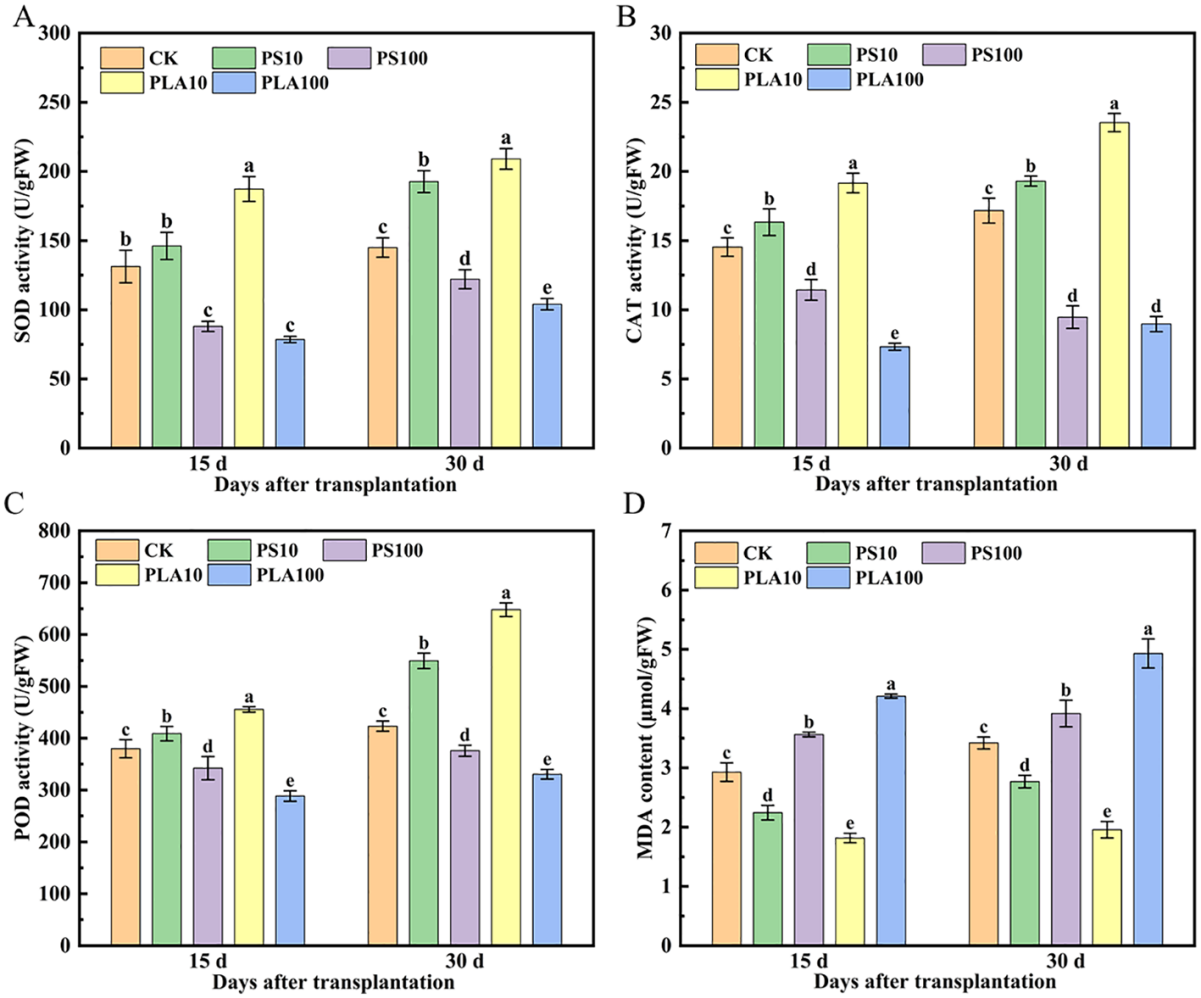
Effects of PS and PLA NPs of different concentrations on antioxidant enzyme activities: **(A)** SOD, **(B)** CAT, **(C)** POD and **(D)** MDA. The bars with different lowercase letters indicate significant differences among the treatments (n=3, *P* < 0.05).

NP treatments can affect the antioxidant enzyme activities of tobacco seedlings (**Figure 4**). At 15 days, superoxide dismutase (SOD) activity under CK and PS10 treatments showed similar levels, with average values of 131.27 and 146.13 U g^-1^, respectively. Compared to CK, the SOD activity decreased by 32.9% and 40.1% under PS100 and PLA100 treatments, respectively. Catalase (CAT) activity increased by 12.4% and 31.9% under PS10 and PLA10 treatments, while it decreased by 21.3% and 49.6% under PS100 and PLA100 treatments, respectively. Peroxidase (POD) activity showed no significant increase under PS10 treatment, while PLA10 treatment showed an increase of 19.9%. POD activity decreased by 10.0% and 24.1% under PS100 and PLA100 treatments, respectively. It is worth noting that the effect of NP treatment on Malondialdehyde (MDA) content is opposite to SOD, CAT and POD activities. MDA content decreased by 23.5% and 37.9% under PS10 and PLA10 treatments, respectively, but increased by 21.5% and 43.7% under PS100 and PLA100 treatments relative to CK. At 30 days, antioxidant enzyme responses showed a similar concentration-dependent trend. Compared to CK, SOD activity increased by 32.8% and 44.2% under PS10 and PLA10 treatments, while it decreased by 15.8% and 28.2% under PS100 and PLA100 treatments, respectively. CAT activity increased by 12.4% and 37.0% under PS10 and PLA10 treatments, respectively, whereas PS100 and PLA100 treatments resulted in comparable CAT levels (9.47 and 8.97 U g^-1^, respectively), both lower than CK. POD activity increased by 29.8% and 53.1% under PS10 and PLA10 treatments, while it decreased by 11.2% and 21.9% under PS100 and PLA100 treatments, respectively. In contrast, compared to CK, MDA content decreased by 19.0% and 42.7% under PS10 and PLA10 treatments, while it increased by 14.3% and 44.2% under PS100 and PLA100 treatments, respectively. Low concentrations of NPs enhanced antioxidant enzyme activities and reduced lipid peroxidation, while high concentrations suppressed enzyme activities and promoted oxidative damage. PLA generally exerted stronger effects on antioxidant enzyme activities than PS at the same concentration.

### Correlation analysis

**Figure 5:**
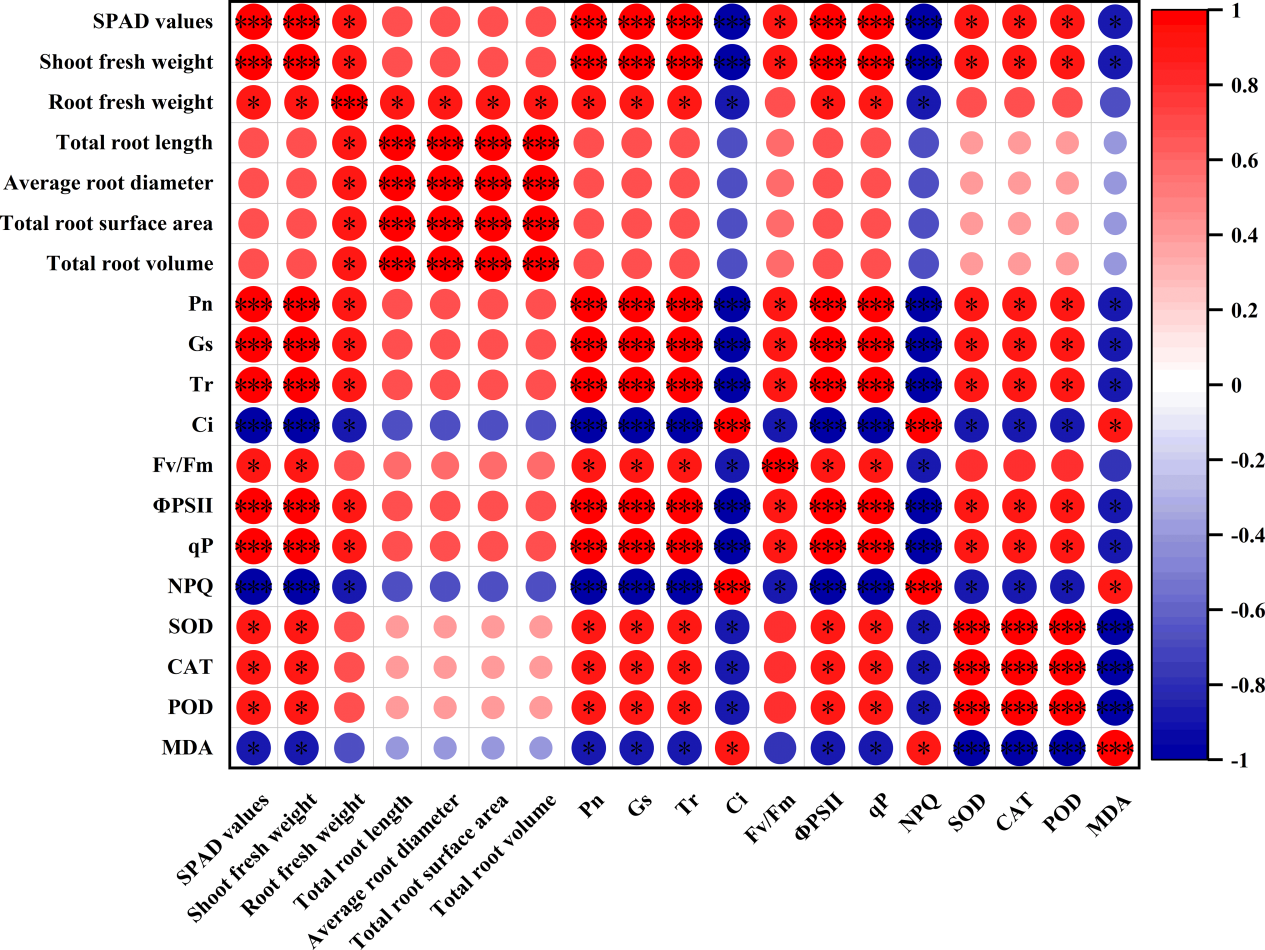
Correlation heat map of tobacco seedling indicators. “*” denotes significant differences (n=3, *P* < 0.05), “**” denotes highly significant differences (n=3, *P* < 0.01), and “***” denotes highly significant differences (n=3, *P* < 0.001).

**Figure 5** presents a Pearson correlation analysis of different variables. The analysis revealed strong associations among growth traits, photosynthetic parameters, root morphological characteristics, and antioxidant enzyme activities in tobacco seedlings under NP treatments. A significant positive correlation existed between SPAD values and shoot and root fresh weight, and they were also positively associated with P_n_, G_s_, and T_r_, indicating that biomass accumulation was closely linked to photosynthetic carbon assimilation capacity. Chlorophyll fluorescence parameters (F_v_/F_m_, ΦPSII, and qP) were likewise significantly positively correlated with gas exchange parameters (P_n_, G_s_, and T_r_), suggesting a close association between photosystem II efficiency and leaf photosynthetic performance under NP stress. Root morphology indicators (e.g., total root length, root surface area, and root volume) were positively associated with shoot growth and photosynthetic performance, indicating that root morphological traits were closely associated with shoot growth and photosynthetic performance. However, root morphology indicators were negatively correlated with NPQ and MDA content, suggesting that NP exposure impairs root function by enhancing energy dissipation and membrane lipid peroxidation. Regarding the antioxidant system, SOD, CAT, and POD activities were significantly positively correlated with one another and negatively correlated with MDA content, indicating coordinated responses among antioxidant enzyme activities and oxidative stress indicators under NP exposure. Overall, the correlation analysis revealed close associations among root morphology, photosynthetic performance, antioxidant enzyme activities, and growth traits in tobacco seedlings exposed to NPs.

### Visual observation of PS and PLA nanoplastics in tobacco seedlings

**Figure 6:**
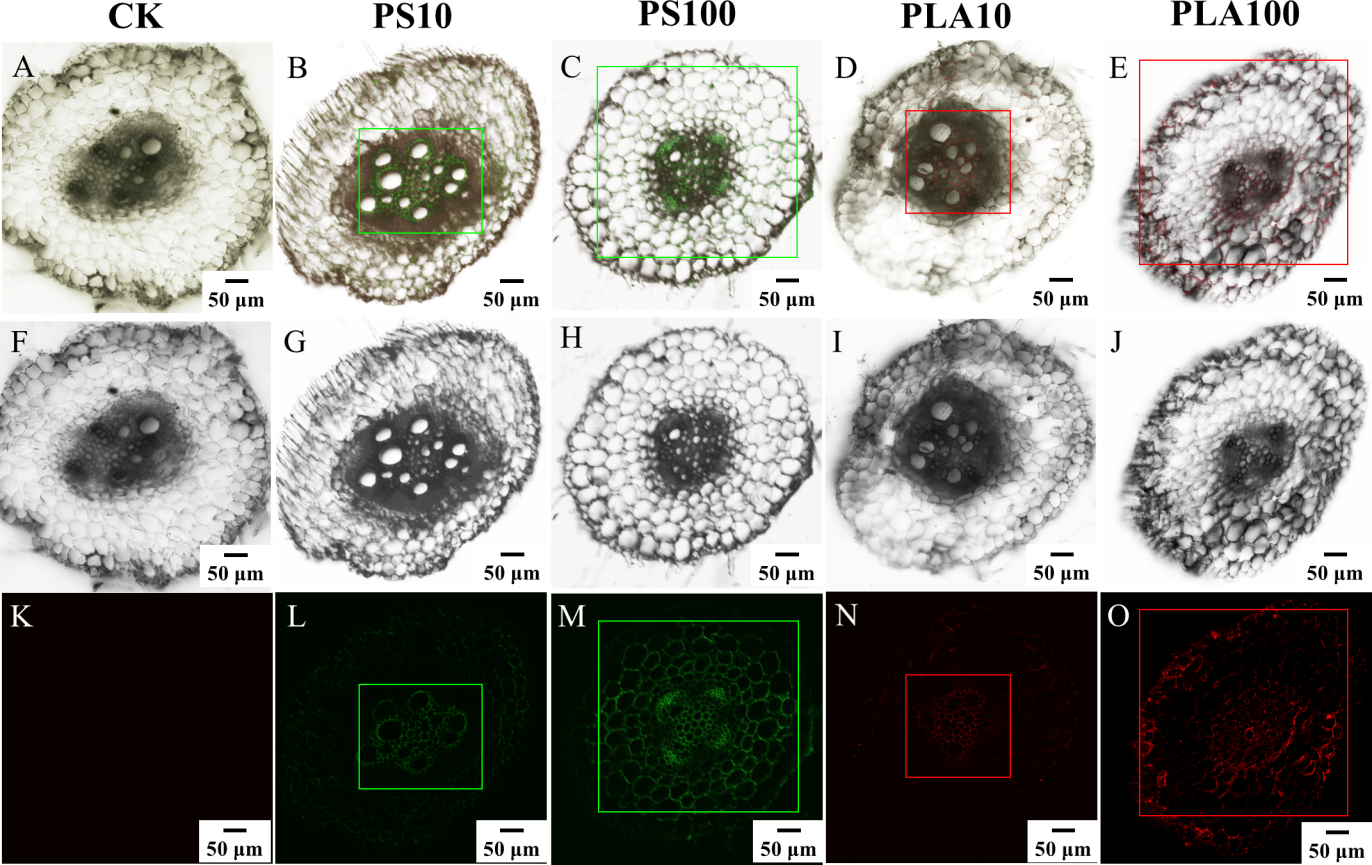
Confocal microscopic images of the tobacco root cross-sections after 30 days of exposure to different treatments. **(A–E)** are the corresponding merged images of bright-field images and fluorescent images; **(F–J)** are bright-field images; and **(K–O)** are fluorescent images.

**Figure 7:**
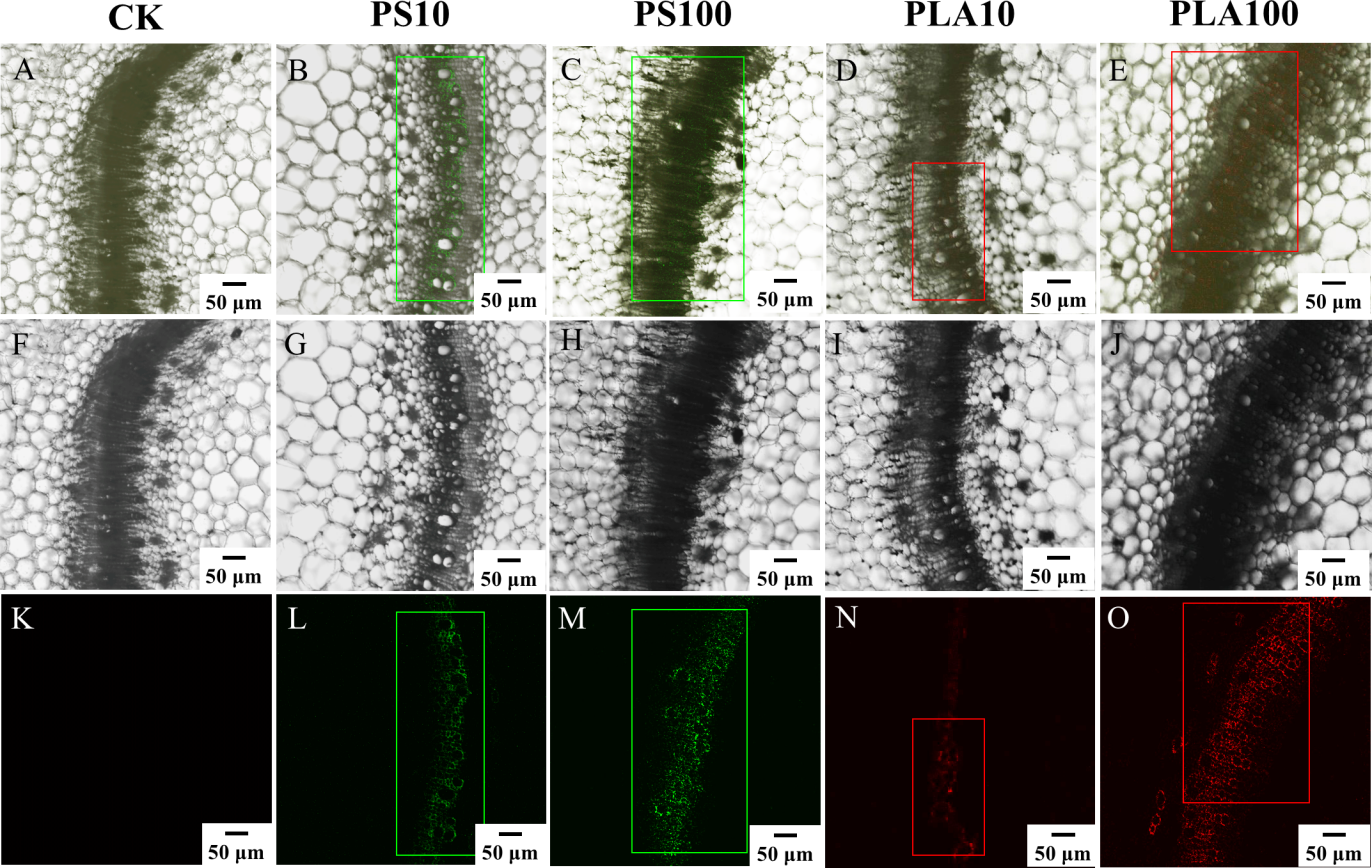
Confocal microscopic images of the tobacco stem cross-sections after 30 days of exposure to different treatments. **(A–E)** are the corresponding merged images of bright-field images and fluorescent images; **(F–J)** are bright-field images; and **(K–O)** are fluorescent images.

**Figure 8:**
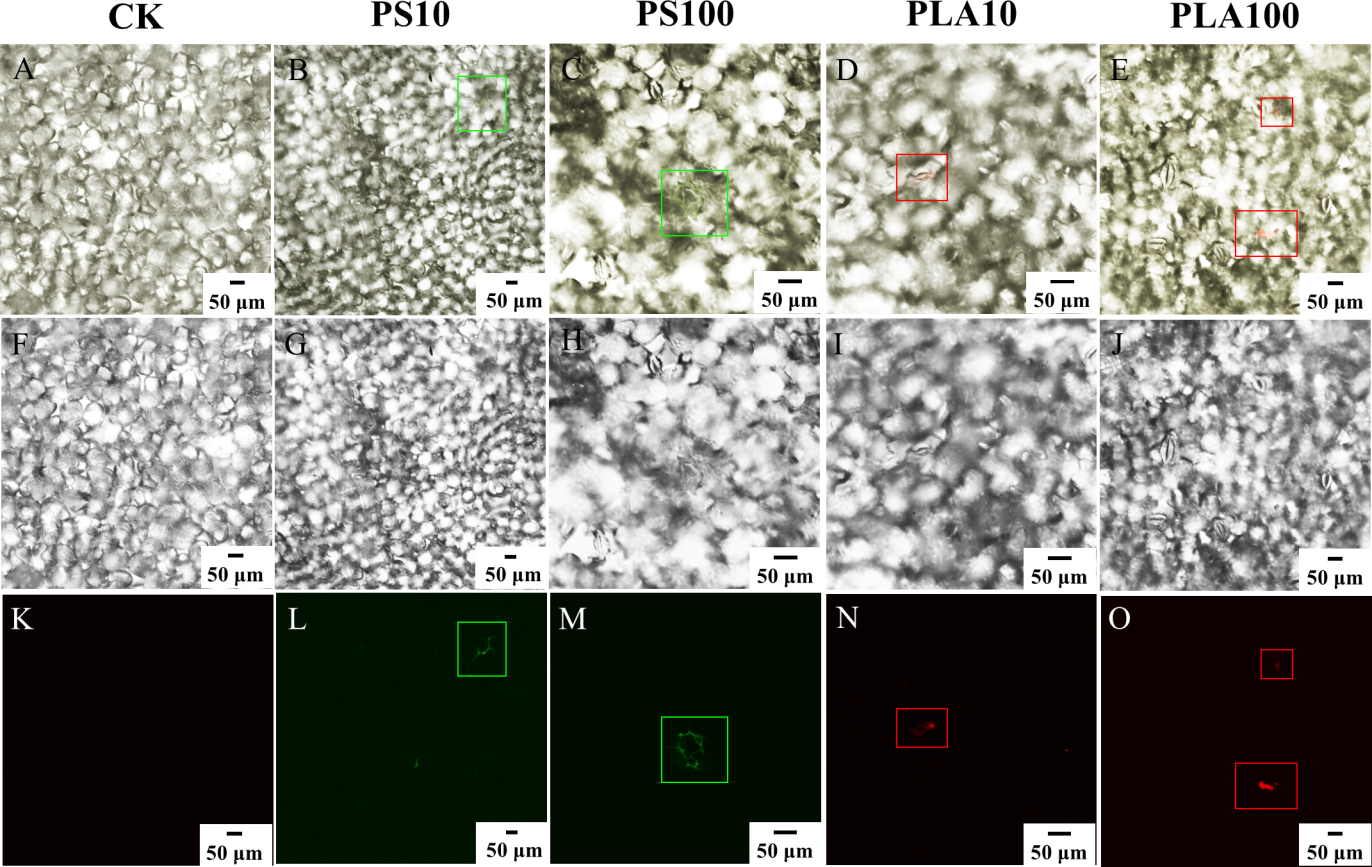
Confocal microscopic images of the tobacco leave slices after 30 days of exposure to different treatments. **(A–E)** are the corresponding merged images of bright-field images and fluorescent images; **(F–J)** are bright-field images; and **(K–O)** are fluorescent images.

After 30 days of hydroponic cultivation, tobacco seedlings were observed using confocal laser scanning microscopy (CLSM) (**Figures 6**–**8**). The control group (CK) showed no detectable fluorescence signals in roots, stems, and leaves, indicating that there was no interference from fluorescent markers under normal conditions. In contrast, fluorescently labeled NP signals were clearly observed in treated plants. At low-concentration treatments, weak fluorescence signals were detected in the roots, stems, and leaves tissues. At high-concentration treatments, fluorescence intensity was markedly enhanced in roots and stems, while fluorescence intensity was only slightly enhanced in the leaves. From the perspective of tissue distribution, fluorescence signals in roots were mainly distributed in the epidermal layer and the central column area, with partial signals appearing in vascular tissues and showing a trend of spreading from the inside outward. The fluorescence signals in stems were mainly detected in the vascular bundle area. In leaves, fluorescence signals were predominantly located in the mesophyll and vascular tissues. These results suggest that the NPs were likely taken up by the roots and then translocated to stems and leaves through vascular tissues. It should be noted that CLSM observations were performed on fresh hand-cut sections without chemical fixation. The images provide qualitative evidence of NP uptake and tissue-level distribution, but subcellular localization cannot be precisely determined.

### FTIR analysis of tobacco seedlings under PS and PLA nanoplastics exposure

**Figure 9:**
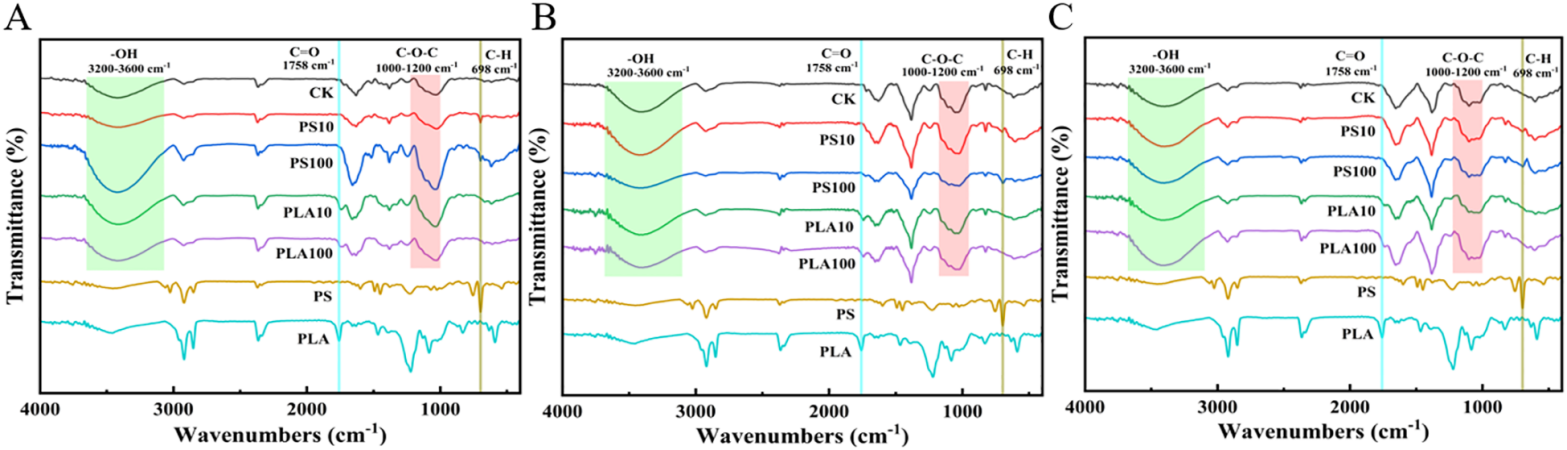
FTIR spectra under different NPs treatments: **(A)** roots, **(B)** stems and **(C)** leaves.

The FTIR spectra of roots, stems, and leaves of tobacco seedlings under different NP treatments are shown in **Figure 9**. The results indicated that in the FTIR spectra of roots, stems, and leaves, the PS-treated samples exhibited a new absorption peak at 698 cm^-1^, corresponding to the out-of-plane bending vibration of the aromatic C-H bond, which is a characteristic peak of PS NPs. The PLA-treated samples displayed a new absorption peak at 1758 cm^-1^, corresponding to the stretching vibration of the ester group C=O bond, which is a characteristic peak of PLA. Moreover, the intensity of these characteristic peaks increased with increasing NP concentration, indicating a concentration-dependent accumulation of NPs in plant tissues. The peak observed in the 3200–3600 cm^-1^ region corresponds to the -OH stretching vibration, and its broadening or intensification typically reflects elevated oxidative stress levels in plants. Compared to CK, PS and PLA treatments exhibited varying degrees of peak broadening and intensity enhancement in the -OH peaks of roots, stems, and leaves, with PS100 treatment showing the most significant changes. Exposure to different concentrations of PS and PLA appears to induce oxidative stress responses in root, stem, and leaf tissues, which aligns with the changes in antioxidant enzyme activities and MDA content (**Figure 4**). Additionally, the peak observed in the 1000–1200 cm^-1^ region corresponds to C-O-C stretching vibrations of cellulose/hemicellulose. Broadening or enhancement of this peak generally suggests possible alterations to plant xylem structure. Compared to CK, PS and PLA treatments caused noticeable broadening and increased intensity of the C-O-C peak in roots and stems, whereas no significant changes were observed in leaves. These findings suggest that different concentrations of PS and PLA may induce chemical modifications in cell wall components associated with vascular tissues in roots and stems.

## Discussion

### Effects of PS and PLA nanoplastics on tobacco seedling growth

Maximum leaf area and fresh weight are important indicators of growth performance and biomass accumulation in tobacco seedlings. This study demonstrated that NP treatment exerted pronounced concentration and type dependent effects on seedling growth, characterized by low-concentration promotion and high-concentration inhibition (**Table 1**). Similar biphasic patterns have been reported under hydroponic conditions in Chinese cabbage and cherry radish exposed to PS microplastics (Chen et al., 2025c). According to Adhikari et al (Adhikari et al., 2025), the slight growth promotion at low concentration treatments may be attributed to reversible cellular stress, which activates the antioxidant defense system, thereby alleviating NP-induced toxicity and maintaining cellular homeostasis. In contrast, high concentration treatments inhibited biomass accumulation, likely due to aggravated oxidative damage, impaired photosynthetic capacity, and disrupted energy metabolism (Nazari et al., 2025; Song et al., 2023; Wu et al., 2026). Fresh weight analysis further confirmed significant concentration and type effects of NPs. Compared to shoots, roots were more sensitive to NP stress, indicating that root tissues are more susceptible under hydroponic conditions. Direct contact between roots and NPs facilitates NPs adsorption and internalization into root, thereby interfering with water and nutrient absorption (Xu et al., 2026; You et al., 2026). Low concentrations of NPs slightly promoted root elongation, which may be attributed to mild stress-induced stimulation of root metabolic activity and enhanced cell division and elongation. Similar responses have been reported by Fu et al. (2023) in studies on Najas natans roots. High-concentrations of NPs inhibited root elongation, possibly due to NP accumulation in root tissues that obstructs apoplastic transport pathways or disrupts cellular structure, ultimately restricting root extension and lateral root formation (Chen et al., 2025a). Differences between NP types are pronounced. At the same concentration, PLA generally exhibited stronger inhibitory effects on growth parameters than PS. Exposure to PS and PLA NPs was associated with changes in root morphology, biomass accumulation, photosynthetic performance, and antioxidant responses in tobacco seedlings. It should be noted that the present study was conducted under hydroponic conditions, which may enhance the bioavailability and root uptake of NPs compared with natural soil environments. In agricultural soils, NP transport and bioavailability are influenced by multiple factors, including soil texture, organic matter content, aggregation, adsorption to soil particles, and microbial activity. Consequently, the inhibiting effects observed in this study may represent a relatively high-exposure scenario and should be interpreted cautiously when extrapolating the results to field conditions.

### Effects of PS and PLA nanoplastics on photosynthetic efficiency of tobacco seedling

Photosynthesis is a critical physiological indicator for evaluating plant physiological status and responses to environmental stress. The SPAD value measurement results (**Figure 1**) revealed that NP exerted concentration-dependent effects on the relative chlorophyll content of tobacco seedlings, with slight increases or remaining stable under low-concentration treatments, while significant decreases under high concentrations. Similar patterns were reported by Li et al. (2020b) in their study on PS NPs in cucumber, where the low concentration promoting effect was attributed to mild stress-induced defense responses, while the high concentration inhibiting effect was associated with chloroplast structural damage and suppressed chlorophyll biosynthesis. Gas exchange parameters further revealed changes in photosynthetic function. The decrease in photosynthetic rate is usually regulated by stomatal factors (characterized by parallel changes in G_s_ and C_i_) or non-stomatal factors (characterized by opposite trends in G_s_ and C_i_) (Chan-juan et al., 2023; Sarabi et al., 2019). The present results (**Figure 2**) showed that P_n_ decreased progressively with increasing NP concentration, with the most pronounced inhibition observed under high-concentration PLA treatment (PLA100). The decrease in G_s_ indicates that stomatal limitation plays a role in the process of photosynthetic decline. Moreover, NP entering the plant tissues may interfere with nutrient transport, reduce chlorophyll content, and induce oxidative damage, thereby further restricting carbon assimilation processes through non-stomatal limitations (Jiang et al., 2019). Chlorophyll fluorescence parameters can sensitively reflect the functional status of the photosynthetic apparatus. In this study, high-concentration NP treatments significantly reduced F_v_/F_m_ and ΦPSII of tobacco seedlings (**Figure 3**), indicating damage to PSII reaction centers and decreased photochemical efficiency (Wang et al., 2020). This finding is consistent with the report by Zhu et al (Zhu et al., 2023), who demonstrated that NPs significantly inhibited photosynthesis in rice leaves. Additionally, elevated NPQ under high-concentration treatments suggests enhanced non-photochemical quenching to dissipate excess excitation energy and mitigate photodamage (Dong et al., 2020; Li et al., 2021). Compared to PS, PLA treatments induced greater alterations in fluorescence parameters, implying more severe disruption of photosystem structure and energy transfer processes. This observation agrees with Qi et al (Qi et al., 2018), who reported strong toxicity of biodegradable NPs, further supporting that NPs inhibit tobacco seedling growth by affecting the stability of the photosystem.

### Effects of PS and PLA nanoplastics on antioxidant system of tobacco seedling

The antioxidant system is an important defense mechanism by which plants respond to abiotic stress (Feng et al., 2020; Radharamanan et al., 2025). In this study, the activities of SOD, POD, and CAT were moderately increased under low-concentration treatments (**Figure 4**), indicating activation of the antioxidant defense system to maintain cellular redox homeostasis. However, high-concentration exposure suppressed the activities of these enzymes, which may be attributed to excessive accumulation of reactive oxygen species (ROS), resulting in structural damage to antioxidant enzyme proteins or inhibition of their synthesis (Ahammed et al., 2024). A similar response pattern has been reported in maize exposed to PS-NH_2_ NPs (Sun et al., 2021). Excessive ROS can attack membrane phospholipids and initiate lipid peroxidation, leading to the formation of MDA (Gao et al., 2026). MDA content is widely recognized as a reliable indicator of membrane oxidative damage and stress-induced cytotoxicity (Ghafori et al., 2026). As shown in **Figure 4**, both high-concentration treatments (100 mg L^-1^ NPs) significantly increased MDA accumulation, further confirming that oxidative stress was aggravated when the antioxidant defense system was suppressed (Pehlivan and Gedik, 2021). PLA NPs induced more pronounced oxidative damage than PS NPs at the same concentration, highlighting differences among NP types in their capacity to trigger oxidative stress. Notably, the concentrations used here are higher than typical environmental levels, and the 100 mg L^-1^ results represent a worst-case scenario with potential non-specific stress responses.

### Distribution and spectral characterization of PS and PLA nanoplastics in tobacco seedlings

Plants can absorb and transport nanoplastics (NPs) through multiple pathways, including surface adsorption and apoplastic transport after root uptake (Gao et al., 2025; Liu et al., 2025b). Confocal laser scanning microscopy (**Figures 6**–**8**) showed that fluorescently labeled NPs were localized in the root epidermis and stele, as well as in the vascular bundles of stems and leaves of tobacco seedlings. NPs may initially be absorbed by the root system, then transported upward through vascular tissues such as the xylem, and eventually distributed to stems and leaves. Similar translocation pathways were reported by Lian et al. (2020) in rice. The internalization and translocation of NPs may physically block cell wall pores and restrict water and nutrient transport, while also triggering cellular damage, membrane lipid peroxidation, and reduced photosynthetic efficiency, thereby disturbing physiological homeostasis and ultimately leading to decreased plant biomass (Arshad et al., 2025; Li et al., 2020a). This study prepared cross-sections, which can visualize the accumulation of NP in different tissue types. Longitudinal sections were not performed, and thus continuous NP movement along the entire root or stem length could not be directly evaluated. Future studies could include longitudinal sectioning for a more detailed analysis of NP translocation.

FTIR analysis provides insight into the underlying mechanisms of NP-induced stress (**Figure 9**). FTIR analysis indicated that PS (698 cm^-1^) and PLA (1758 cm^-1^) characteristic peaks appeared in roots, stems, and leaves, with intensities increasing with NP concentration, reflecting progressive accumulation of NPs in plant tissues. Slight broadening and shifting of the -OH stretching band (3200–3600 cm^−^¹) may suggest disturbance of cellular redox homeostasis, consistent with changes in antioxidant enzyme activities and MDA content. Modifications in the C-O-C region (1000–1200 cm^-1^) imply potential alterations in cellulose and vascular structures, which may affect water transport and photosynthetic performance. These spectral changes indicate that NPs influence tobacco physiology through combined effects of tissue accumulation, oxidative stress induction, and subtle interactions with cellular components.

### Potential mechanism analysis

**Figure 10:**
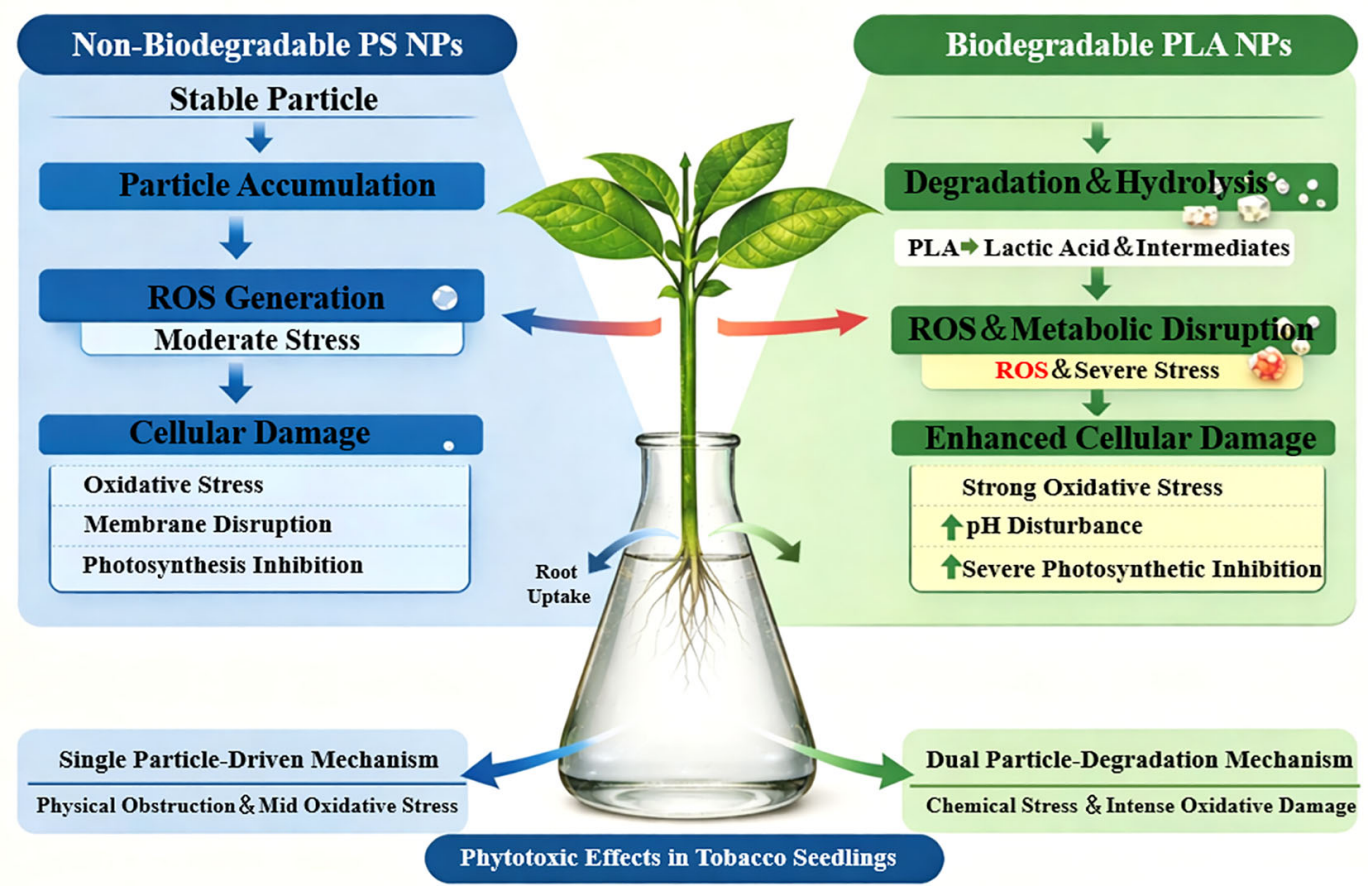
Potential mechanism analysis of the phytotoxic effects of PS and PLA NPs on tobacco seedlings.

The phytotoxic effects of NPs on tobacco seedlings are primarily governed by their physicochemical properties, which may lead to distinct response mechanisms between biodegradable PLA and non-biodegradable PS NPs (**Figure 10**). Although both types of NPs have similar uptake and translocation pathways—being absorbed by roots and further transported to the stems and leaves, their modes of action after internalization may differ substantially. For non-biodegradable PS NPs, the dominant mechanism may be particle-driven toxicity. Due to its chemically stable and hydrophobic aromatic structure, PS is resistant to degradation within plant tissues and mainly accumulates in roots and vascular bundles (**Figures 6**–**8**). This accumulation may lead to physical obstruction effects (partial blockage of cell wall pores and xylem conduits), thereby restricting water and nutrient transport (Chen et al., 2024; Zhu et al., 2022). In addition, PS NPs can induce moderate levels of ROS, triggering oxidative stress (**Figure 4**). Combining our observations and previous studies, we speculate that ROS production at low NP concentrations activates antioxidant defense systems and promotes plant growth, while excessive ROS accumulation at high NP concentrations leads to oxidative damage, enzyme inhibition and impaired photosynthetic efficiency. However, because PS lacks degradability and strong chemical reactivity, its effects are relatively gradual, and the ROS generation may mainly be associated with physical interference and particle induction. In contrast, biodegradable PLA NPs may exhibit a more complex dual-effect mechanism, involving both particle toxicity and degradation-induced chemical stress. Previous studies have suggested that PLA undergo partial hydrolysis after entering biological systems due to the presence of ester bonds (C=O), generating low-molecular-weight intermediates such as lactic acid and oligomers (An et al., 2025). These degradation products have been proposed to influence intracellular physiological processes and contribute to cellular stress responses (Chen et al., 2025b). Moreover, the relatively higher polarity and surface reactivity of PLA may facilitate stronger interactions with plant cell wall components, particularly cellulose and hemicellulose, leading to more pronounced structural alterations, as supported by FTIR changes in the C-O-C region (**Figure 9**). This enhanced interaction likely intensifies plant vascular interference and restricts transport processes more severely than PS. Importantly, PLA induces significantly stronger oxidative stress responses than PS. The combined effects of particle accumulation and potential degradation-derived intermediates may contribute to enhanced oxidative stress responses, which rapidly overwhelm the antioxidant defense system, leading to increased membrane lipid peroxidation, reduced enzyme activities, and severe damage to chloroplast structure and photosystem II (**Figures 3**, **4**). Overall, based on previous literature and the physiological responses observed in this study, the mechanisms of the two NPs can be summarized as follows: PS may exert toxicity through a single particle-driven pathway characterized by physical accumulation and moderate oxidative stress, whereas PLA toxicity may involve both particle-related effects and potential degradation-associated processes. This proposed mechanistic difference may explain the stronger inhibitory effects of PLA observed in this study and highlights that biodegradable NPs may pose greater ecological risks than previously considered inert, non-biodegradable counterparts. The degradation behavior of PLA and the physiological effects of its degradation products were not directly measured in the present study. The proposed degradation-associated toxicity pathway should be regarded as a hypothesis requiring further verification.

## Conclusions

This study systematically investigated the uptake, translocation, and phytotoxic effects of PS and PLA NPs in tobacco seedlings under hydroponic conditions, and further elucidated their distinct phytotoxic mechanisms. Both PS and PLA NPs were detected in roots, stems, and leaves, exhibiting concentration-dependent accumulation. Their phytotoxicity was primarily associated with a cascade mechanism involving NPs accumulation, oxidative stress induction, and photosynthetic system disruption, accompanied by structural modifications in cellulose-related components. Excessive accumulation of NPs triggered reactive oxygen species (ROS) overproduction, leading to redox imbalance, membrane lipid peroxidation, and impairment of photosystem II efficiency. In the potential mechanism, distinct differences were observed between PS and PLA. PS NPs primarily exerted toxicity through a particle-driven mechanism characterized by physical accumulation and moderate oxidative stress. In contrast, PLA NPs exhibited a dual-effect mechanism, combining particle toxicity with degradation-induced chemical stress, which enhanced ROS generation and aggravated intracellular disturbance. As a result, PLA caused more severe oxidative damage and photosynthetic inhibition than PS at equivalent concentrations. Overall, these findings demonstrate that biodegradable NPs may pose greater ecological risks than non-biodegradable NPs, providing new insights into nanoplastic-plant interactions and a scientific basis for evaluating their impacts on agricultural systems. Future studies should validate these findings under realistic soil conditions to better assess the environmental risks of NPs in agricultural ecosystems.

## Data Availability

The original contributions presented in the study are included in the article/Supplementary Material. Further inquiries can be directed to the corresponding authors.
